# Comment on ‘Unexpected plasticity in the life cycle of *Trypanosoma brucei*’

**DOI:** 10.7554/eLife.74985

**Published:** 2022-02-01

**Authors:** Keith R Matthews, Stephen Larcombe

**Affiliations:** 1 Institute for Immunology and Infection Research, School of Biological Sciences, University of Edinburgh Edinburgh United Kingdom; DKFZ-ZMBH Alliance Germany; University of New South Wales Australia

**Keywords:** *Trypanosoma brucei*, transmission, stumpy form, parasite, *Trypanosoma congolense*, tsetse fly, Other

## Abstract

Schuster et al. make the important observation that small numbers of trypanosomes can infect tsetse flies, and further argue that this can occur whether the infecting parasites are developmentally ‘slender’ or ‘stumpy’(Schuster et al., 2021). We welcome their careful experiments but disagree that they require a rethink of the trypanosome life-cycle. Instead, the study reveals that stumpy forms are more likely to successfully infect flies, the key limit on parasite transmission, and we predict this advantage would be greatly amplified in tsetse infections in the field. Further, we argue that stumpy forms are defined by a suite of molecular adaptations for life-cycle progression, with morphology being a secondary feature. Finally, their dominance in chronic infections means most natural tsetse infections would involve stumpy forms, even in small numbers. Our interpretation does not require re-evaluation of the obligatory life cycle of the parasite, where stumpy forms are selected to sustain transmission.

## Introduction

In the bloodstream of mammalian hosts trypanosomes proliferate as morphologically slender forms and undergo density-dependent quorum sensing in response to oligopeptides generated by parasite-released peptidases (Rojas et al., 2019). In response, the parasites undergo cell cycle arrest and morphological transformation to stumpy forms. Stumpy formation has been proposed to serve two functions for the parasite (Matthews, 2021). Firstly, their growth arrest prevents parasite numbers overwhelming the host and so prolongs the infection and thus probability of transmission. Secondly, stumpy forms are adapted for transmission, both being more able to survive uptake by tsetse flies (Turner et al., 1988) and by expressing molecules necessary for onward development. Notable among these are PAD family proteins that sense the change in environment upon entering the fly and stimulate parasite differentiation (Dean et al., 2009). Several signalling molecules expressed by stumpy forms are also important for the initiation of parasite differentiation, as are RNA regulatory proteins (Walsh and Hill, 2021). Further, as the cells initiate differentiation, they are protected from complement in the blood meal by the expression of the factor H receptor, which is enriched in stumpy forms (Macleod et al., 2020). Finally, classic ultrastructural (Vickerman, 1965) and recent biochemical studies (Dewar et al., 2018) demonstrate that stumpy forms elaborate their mitochondrion and exhibit metabolic pre-adaptation for the tsetse midgut environment. In combination, this suite of molecular adaptations prepares the parasite for development in the fly.

In their recent article, Schuster et al. explored tsetse infectivity by slender and stumpy forms through a challenging series of experiments involving the infection of tsetse by limited numbers of parasites (Schuster et al., 2021). They then dissected the flies after parasite establishment to analyse the progression of infection from establishment in the midgut, and subsequent migration to the proventriculus and on to the salivary glands where mammalian infectivity is regained. For these experiments, trypanosomes were used that were either morphologically slender and replicative or were stumpy and arrested. The stumpy forms were derived either through exposure to ‘stumpy induction factor’ representative of accumulated oligopeptides in parasite conditioned medium, or through engineered ectopic expression of a second VSG gene – this having been previously shown to provoke parasite cell cycle arrest and stumpy formation (Zimmermann et al., 2017). Interestingly, even a very low infective dose of parasites could result in fly infection and either slender or stumpy forms could result in mature salivary gland parasite populations.

Further examination of the kinetics of infection by the parasites revealed that stumpy cells expressed PAD1 at the outset of infection (as expected) whereas slender forms progressively expressed PAD1 in the population over 24 hr in the tsetse midgut. Thereafter a surface coat protein of the insect stage of the parasite, EP procyclin, was expressed – this occurring rapidly in the stumpy-derived infections and progressively in slender-derived infections following PAD1 expression. No evidence of an extended cell cycle arrest – as observed for stumpy forms – was seen for the slender forms that transformed in these tsetse midguts. In combination, a re-evaluation of the parasite life cycle was proposed such that tsetse infections could be generated by either slender or stumpy forms with similar efficiency, challenging the transmission adaptation of stumpy forms held in the existing literature. This apparent equivalence in infectivity is also proposed to resolve a ‘transmission paradox’, whereby insufficient numbers of stumpy cells in infected animals might make transmission unlikely.

## Results and discussion

### Evidence for the importance of stumpy forms

We offer an alternative interpretation for these experiments, retaining the importance of stumpy forms in the cyclical transmission of *Trypanosoma brucei*. This is based on a number of points that we summarise as follows.

**Figure 1.**
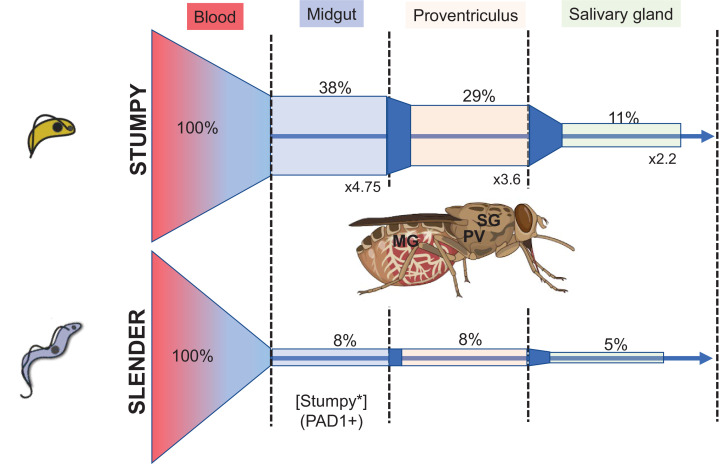
Trypanosomes establish tsetse fly infections better when stumpy rather than when slender in morphology. The proportion of flies infected from a bloodstream population of two slender or two stumpy forms is shown, with % of infections represented and the fold difference in % infection between stumpy and slender initiated feeds represented. The bars are to scale with respect to the % infection, with the 100% set as the proportion of flies fed. Data derived from Schuster et al., involving stumpy forms induced by quorum sensing. Tsetse fly image generated by BioRender - biorender.com.

1. The data of Schuster et al., and others (Van Den Abbeele et al., 1999), demonstrates that a key bottleneck for transmission is initial infection of the fly, as well as maturation once established (Oberle et al., 2010). Regardless of parasite dose, fly infection is inefficient but naturally induced stumpy forms are considerably more successful than slender forms ( > 4 fold for SIF-induced parasites; 38.8% vs 7.8% midgut infections from Figure 2 of Schuster et al.). Instead, Schuster et al. argue that the maturation from midgut infection to salivary gland infection is most biologically relevant and make the interesting observation that slender derived infections more effectively progress from the midgut to salivary gland than stumpy forms (generating a higher ‘transmission index’). However, the transmission index is subject to the phenomenon of ‘survivorship bias’ (i.e. it does not account for most instances, where parasites do not survive to establish a midgut infection in the first place) and when considering all flies for which infection was attempted, SIF-induced stumpy forms establish twice as many mature salivary gland infections as slender forms from the same infective dose (Figure 1). Indeed, with respect to both initial infectivity and overall maturation, stumpy forms perform better.
2. The ability of slender forms to transition to stumpy forms in the fly gut has been reported (Wijers and Willett, 1960), if parasites can survive long enough to do so. This could be stimulated by gut oligopeptides activating the quorum sensing pathway, or the stress of the midgut environment (Quintana et al., 2021). Parasite development in the insect gut is possible in the flies used by Schuster et al, which were recently eclosed (hatched) and so lacked a mature immune system that characterises the resistance of adult flies to trypanosome infection (Haines, 2013). Inclusion of N-acetylglucosamine in the feed also reduces lectin-based defences of the tsetse and potentially alters trypanosome metabolic adaptation (Peacock et al., 2006), whereas the lysis by complement of slender forms lacking the factor H receptor is avoided by the absence of a rapid second feed (Macleod et al., 2020). These are experimentally routine approaches to successfully establish trypanosome infections but they generate an unnaturally permissive environment where slender cells can persist. As noted above, even here, infections with slender forms are less able to establish infections than stumpy forms.
3. We argue that stumpy forms in *T. brucei* are defined by their molecular characteristics and not morphology. Hence, regardless of morphology, the expression of the molecular machinery known to be required for life cycle development (e.g. PAD1, TbPTP1, PIP39 etc; Szöor et al., 2020) reflects developmental progression in the fly gut to stumpy forms. These developmentally competent stumpy forms that appear before morphological transformation have been described previously, and termed stumpy* forms (Tasker et al., 2000, Matthews et al., 2004). We note that parasites with reduced ability to generate stumpy forms (monomorphic cells) establish infections in tsetse flies very poorly, although even monomorphic populations retain some cells able to express the molecular characteristics of stumpy forms (Szöor et al., 2006). Overall, we consider the necessity to express PAD1 reported by Schuster et al. reflects an obligatory development to stumpy cells prior to onward differentiation to midgut procyclic forms.
4. The cell cycle arrest of stumpy forms in the mammalian bloodstream controls parasite numbers but has also been proposed as important for their developmental competence (Matthews and Gull, 1994). Schuster et al. note that an extended arrest is not required for development, although a transient cell cycle exit remains possible. This interesting observation is consistent with a recent single-cell RNA analysis of slender to stumpy differentiation, where parasites passed directly from the G1 phase of slender cells into developmentally competent stumpy forms (Briggs et al., 2021). Apparently, in normal stumpy formation, cell-cycle position dependent development occurs but an extended arrest is not required. Alternatively, slender cells may commit to stumpy formation and continue through one or more cell cycles after commitment, a scenario predicted by modelling (MacGregor et al., 2011).
5. Some insight can be gained by considering *Trypanosoma congolense*, an African trypanosome that successfully infects tsetse through establishment in the midgut, as does *T. brucei*, although their ultimate maturation occurs in the mouthparts not the salivary glands (Rotureau and Van Den Abbeele, 2013). Characterised isolates of *T. congolense* do not generate morphologically stumpy forms, potentially supporting the concept that these are not required for transmission in *T. brucei*, and Schuster et al. propose that quorum sensing is an innovation unique to *T. brucei*. However, *T. congolense* does undergo quorum sensing and density dependent arrest in the mammalian host and possesses in its genome the necessary molecular machinery for this (Silvester et al., 2017). Furthermore, through complementation experiments a molecule driving stumpy formation has been shown to be functionally equivalent between species. In combination, this reinforces the view that *T. congolense* can exhibit development in the bloodstream and that morphological parameters are not critical in developmental competence.
6. The transmission paradox invoked by Schuster et al. is predicated on there being insufficient bloodstream stumpy forms in natural infections to sustain infectivity to flies. However, stumpy forms are known to comprise the dominant population in chronic trypanosome infections in the bloodstream (MacGregor et al., 2011), adipose tissue (Trindade et al., 2016) and dermis, the specialised niche believed to contribute to the maintenance of the infective reservoir (Capewell et al., 2016). In each case, stumpy forms can comprise 80% of the parasite population making it likely, if recapitulated in animals in the field, that most parasites transmitted to tsetse would be these forms. Hence, with the infectivity of small numbers of stumpy form parasites observed by Schuster et al., the transmission paradox is resolved (Capewell et al., 2019).

In summary, we consider that the carefully executed experiments of Schuster et al can be interpreted within existing knowledge and without the necessity to challenge the importance of stumpy forms in parasite transmission. The observations that small numbers of trypanosomes can establish infections and then go on to mature is epidemiologically important and supportive of previous studies with *Trypanosoma congolense*, a species that follows a similar establishment path as *T. brucei* in the tsetse (Maudlin and Welburn, 1989). We also emphasise that we agree that slender cells can establish infections in flies in the laboratory conditions used, but with the caveat that they must first become stumpy in the fly to do so.

### Biological perspectives on the importance of stumpy forms in Trypanosome transmission

There are the central biological questions that emerge from this thought-provoking study. If stumpy forms are unnecessary for transmission, why are they maintained at a fitness cost to parasites in the field? Without tsetse transmission, laboratory lines rapidly reduce stumpy formation but pleomorphism is sustained in the field. This would not be expected if slender and stumpy forms can infect flies with equivalent efficiency. Further, if stumpy forms are not important for transmission, then why do the *Trypanosoma brucei* subspecies *T. b. evansi*, and *T. b. equiperdum*, which are no longer transmitted by tsetse flies, invariably lose the ability to generate stumpy forms and become monomorphic?

Overall, a simple role for stumpy forms in population control in the mammalian host is incompatible with the sophisticated molecular adaptations for development and life in the tsetse fly they exhibit. Moreover, in the field the likelihood of parasite transmission is low given the poor infectivity of parasites to tsetse flies. Here, the transmission advantage of stumpy forms provided by their molecular adaptations for onward development in the fly provides the evolutionary benefit that sustains their key importance in the life cycle of the parasite.

## Data Availability

Not applicable.
